# Perivascular Tumor-Infiltrating Leukocyte Scoring for Prognosis of Resected Hepatocellular Carcinoma Patients

**DOI:** 10.3390/cancers10100389

**Published:** 2018-10-18

**Authors:** Markus Bo Schoenberg, Jingcheng Hao, Julian Nikolaus Bucher, Rainer Christoph Miksch, Hubertus Johann Wolfgang Anger, Barbara Mayer, Julia Mayerle, Jens Neumann, Markus Otto Guba, Jens Werner, Alexandr V. Bazhin

**Affiliations:** 1 Department of General, Visceral, and Transplant Surgery, Ludwig-Maximilians-University Munich, Marchioninistraße 15, 81377 Munich, Germany; markus.schoenberg@med.uni-muenchen.de (M.B.S.); haojc416@gmail.com (J.H.); julian.bucher@med.uni-muenchen.de (J.N.B.); rainer.miksch@med.uni-muenchen.de (R.C.M.); hubertusanger@gmail.com (H.J.W.A.); barbara.mayer@med.uni-muenchen.de (B.M.); markus.guba@med.uni-muenchen.de (M.O.G.); jens.werner@med.uni-muenchen.de (J.W.); 2 Department of Hepatobiliary Surgery, The First Affiliated Hospital of Chengdu Medical College, Chengdu 610513, China; 3 Department of Medicine II, University Hospital, Ludwig-Maximilians-Universität Munich, Marchioninistraße 15, 81377 Munich, Germany; Julia.mayerle@med.uni-muenchen.de; 4 Institute of Pathology, Faculty of Medicine, Ludwig-Maximilians-University Munich, Marchioninistraße 15, 81377 Munich, Germany; jens.neumann@med.uni-muenchen.de; 5 Transplantation Center Munich, Ludwig-Maximilians-University Munich, Marchioninistraße 15, 81377 Munich, Germany; 6 German Cancer Consortium (DKTK), Partner Site Munich, Pettenkoferstraße 8a, 80336 Munich, Germany

**Keywords:** tumor-infiltrating leukocytes, hepatocellular carcinoma, quantification of the tumor immune stroma (QTiS), immunology, liver resection

## Abstract

Liver resection is a curative treatment for hepatocellular carcinoma (HCC). Tumor-infiltrating leukocytes (TILs) are important players in predicting HCC recurrence. However, the invasive margin could not be confirmed as relevant for HCC. The migration of immune cells into HCC may originate from intratumoral vessels. No previous study has examined perivascular (PV) infiltration. Tumors from 60 patients were examined. Immunohistochemistry was performed against CD3, CD8, CD20, and CD66b. TILs were counted in the PV regions using an algorithm for quantification of the tumor immune stroma (QTiS). The results were correlated with overall (OS) and disease-free survival (DFS), clinical parameters, and laboratory values. PV infiltration of TILs was predominant in resected HCC. Higher PV infiltration of CD3^+^ (*p* = 0.016) and CD8^+^ (*p* = 0.028) independently predicted better OS and DFS, respectively. CD20^+^ showed a trend towards better DFS (*p* = 0.076). Scoring of CD3^+^, CD8^+^, and CD20^+^ independently predicted OS and DFS (*p* < 0.01). The amount of perivascular-infiltrating CD3^+^ cells is an independent predictor of better OS, and CD8^+^ cells independently predict prolonged DFS. Our novel perivascular infiltration scoring (PVIS) can independently predict both DFS and OS in resected HCC patients.

## 1. Introduction

Hepatocellular carcinoma (HCC) is the most common type of primary liver cancer, accounting for about 70% to 90% of cases. In terms of incidence, it ranks fifth in men and ninth in women and is the second leading reason of cancer death globally [1]. Surgery, including liver resection (LR) and liver transplantation (LT), remains the most effective curative treatments of HCC. It is reported that only 30% of diagnosed HCC patients are suitable for surgery (LR and LT) [2]. Even though outcomes vary widely, approximately 70% of patients undergoing LR [3] and 20% of LT recipients [4] develop recurrence within five years after surgery. Due to this fact, as well as the relative scarceness of organs for LT, careful and meticulous risk stratification is needed to treat patients with the appropriate method. Factors predicting better postoperative survival have been investigated over decades in several aspects including liver function, tumor stage, tumor molecular biology, and lastly, tumor immunology [5,6].

The alterations of number and function of different cell types, cytokine, and chemokine levels have been studied both clinically and experimentally in HCC [7]. In several studies, tumor-infiltrating leukocytes (TILs), especially lymphocytes such as CD3^+^ and CD8^+^ cells, have shown predictive values for better survival [8,9,10,11,12,13,14,15]. However, information about B-cells and innate immune cells (such as neutrophils) is very limited and unclear [10,14,16]. These findings are also similar in several other solid human cancer types, including colorectal cancer [17,18], ovarian cancer [19,20], pancreatic cancer [21], and breast cancer [22,23]. Moreover, independent from the type and the number of TILs, the location of immune cells needs to be considered when predicting the outcome. Three main locations, intratumoral (IT), stromal, and invasive margin (IM), have been defined by previous studies in different entities. Moreover, studies have suggested that high immune cell densities at the IM might have a better prognostic value than IT leukocytes [24,25]. However, in HCC, studies could not confirm that the IM is equally relevant [12,26]. This lack of a difference might be explained by the fact that HCC shows expansive growth within a protective fibrous capsule instead of an infiltrative pattern. Additionally, most HCCs develop in cirrhosis which is also accompanied by an immunosuppressive environment [27,28].

To identify the most relevant battlefront between the immune system and the HCC cells, the largest surface area should be regarded; the surface area of the intratumoral vessels represents the largest border [29]. HCC is predominantly neovascularized with arteries [30]. The formation of these new vessels is essential for tumor growth and progression by providing nourishment and oxygen [31]. Conversely, it can also promote immune cell infiltration which is indeed dependent on the network of blood vessels. It should be stressed that there is evidence that a high degree of endothelial venules within solid human tumors is a strong predictor of the infiltrating T and B cells [32]. In this setting, the migration of immune cells into HCC may not originate from the IM but from extravasation in the intratumoral vessels [33]. Nevertheless, no previous study has provided any information on perivascular infiltration.

Therefore, our study aimed to investigate the interlude between perivascular TILs and overall as well as disease-free survival in patients that received LR because of HCC. The leukocyte markers were chosen to represent the cellular, humoral, and innate immunity.

## 2. Results

### 2.1. Analysis of Epidemiological and Clinicopathological Data of the Study Patients

**Figure 1:**
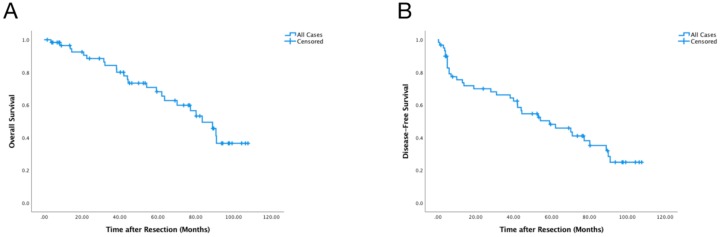
Overall (**A**) and Disease-free Survival (**B**) of All Patients.

Tissue samples from 60 resected HCC tumors were assessed. Most (81.67%) of the patients were male. The median age was 66.00 (16.00) years old. Only seven patients had hepatitis B in their medical history, which was representative for a western European cohort. In our laboratories, hepatitis C tissue was not handled for reasons of worker protection. Therefore, no hepatitis C infection was present in the study cohort. According to the preoperative imaging and postoperative pathological reports, nine patients (15.00%) had more than one tumor lesion. Eighteen cases (30.00%) had microvascular invasions, and six patients (10.00%) showed macrovascular invasions on imaging. Forty-seven (78.33%) were outside the Milan criteria. The demographical characteristics are summarized in Table 1. The median follow-up after resection was 51.2 months. The estimated cumulative proportion of overall survival at 1, 3, 5, and 8 years was 96.4%, 84.2%, 68.1%, and 36.5%; disease-free survival was 75.5%, 66.2%, 48.1%, and 24.8% respectively. (Figure 1) The median overall and disease-free survival times were 83.5 and 59.0 months, respectively. During the follow-up period, 23 (38.33%) patients had recorded recurrence. Seventeen (73.91%) of all patients that suffered recurrence received postoperative treatments for recurrence. The postoperative treatments included transarterial chemoembolization (TACE) (8 cases, 47.06%), Sorafenib (7 cases, 41.18%), repeat hepatectomy (4 cases, 23.53%), selective internal radiation therapy (SIRT) (2 cases, 11.76%), radiofrequency ablation (RFA) (2 cases, 11.76%), and transplantation (1 case, 5.88%). Therefore, our patient cohort was representative of a dynamic multimodal therapy of patients after resection for HCC [5,13].

### 2.2. HCC Infiltration with Leucocytes around the Intratumoral Vessels Is Predominant 

**Figure 2:**
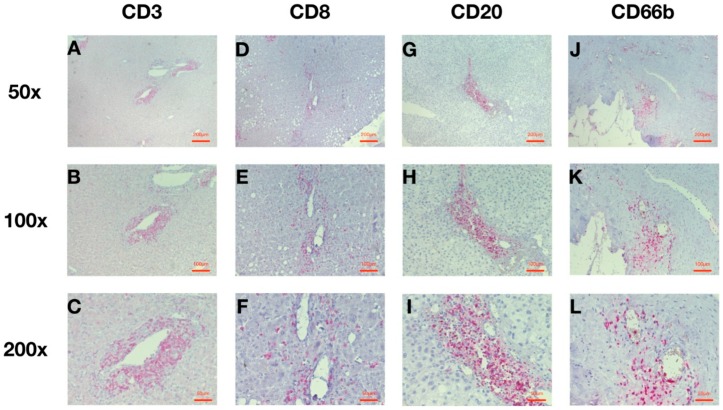
Representative Perivascular Patterns of CD3^+^, CD8^+^, CD20^+^, and CD66b^+^ Cells under 50×, 100×, and 200× Magnifications. (**A**) CD3 at 50× magnification; (**B**) CD3 at 100× magnification; (**C**) CD3 at 200× magnification; (**D**) CD8 at 50× magnification; (**E**) CD8 at 100× magnification; (**F**) CD8 at 200× magnification; (**G**) CD20 at 50× magnification; (**H**) CD20 at 100× magnification; (**I**) CD20 at 200× magnification; (**J**) CD66b at 50× magnification (**K**) CD66b at 100× magnification; (**L**) CD66b at 200× magnification.

Immunohistochemistry analysis revealed that there were three representative patterns of infiltration: (a) Completely negative without any recognized staining (Figure S1), (b) Infiltration evenly distributed (Figure S2), (c) Infiltration located around the intratumoral vessels. (Figure 2). The perivascular infiltration (PVI) patterns were found in most cases among CD3, CD8, CD20, and CD66b (Figure 2 and Table S1).

Due to the distinct patterns as mentioned above, the perivascular regions were examined in detail. The PVI of CD8^+^ cells strongly correlated with CD20^+^ cells (r = 0.856, *p* < 0.001). As expected, significant but weak correlations were also found between the PVI of CD3^+^ cells and CD8^+^ cells (r = 0.375, *p* = 0.003), as well as between the PVI of CD3^+^ cells and CD20^+^ cells (r = 0.487, *p* < 0.001). However, the PVI of CD66b^+^ cells did not correlate with CD3^+^ (r = −0.016, *p* = 0.905), CD8^+^ (r = −0.028, *p* = 0.833), or CD20^+^ (r = −0.071, *p* = 0.589) lymphocytes (Figure S3).

A survival analysis of the three patterns of infiltration revealed no significant difference in OS for any marker used in this study. When analyzing disease-free survival (DFS), an influence on survival could be detected for the infiltration patterns of CD8 and CD20. Perivascular infiltration of CD8^+^ cells showed significantly better survival than evenly distributed infiltration and no infiltration (*p* = 0.049). In the case of CD20^+^ infiltration, no infiltration had significantly worse DFS compared to evenly distributed and perivascular infiltration (*p* = 0.027).

### 2.3. PVI CD3^+^ and CD8^+^ Cells Predict DFS of HCC Patients 

For Kaplan-Meier survival analysis, all patients were divided into high or low infiltration groups as described in the material and methods section. In this cohort, we defined high PVI for CD3^+^ from 21 counted cells. The cut-point for high PVI of CD8^+^ and CD20^+^ was 10, and high PVI of CD66b^+^ was 9 counted cells. The median cell-density for CD3^+^, CD8^+^, CD20^+^, CD66b^+^ were 276 cells/mm^2^, 72 cells/mm^2^, 69 cells/mm^2^, 62 cells/mm^2^, respectively.

Regarding demographic and routine laboratory data, almost no differences were found between high and low perivascular infiltration of leukocytes. Only albumin was lower in the case of high PVI of CD3^+^ and CD8^+^ (Table S2).

**Figure 3:**
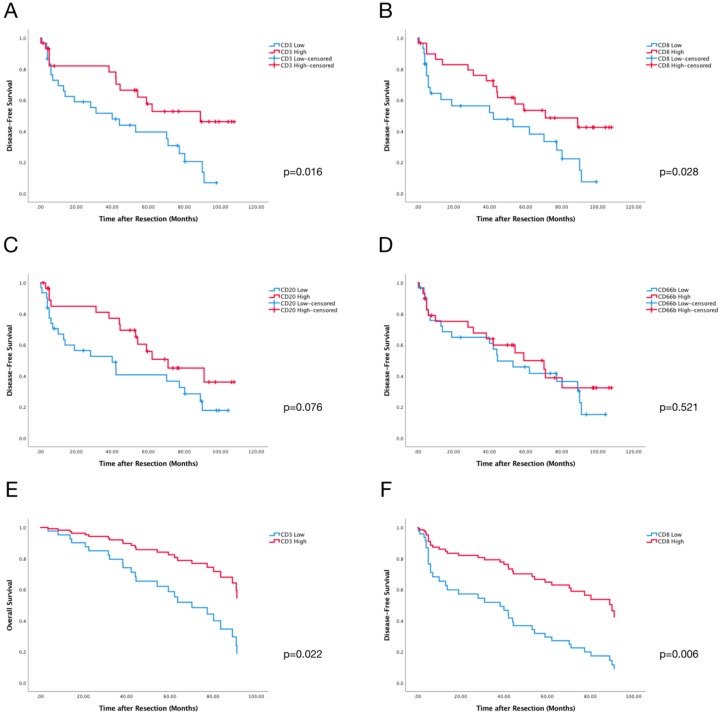
Kaplan-Meier Curves of CD3^+^ (**A**), CD8^+^ (**B**), CD20^+^ (**C**), and CD66^+^ (**D**) cells for DFS. (**E**) Cox Regression Curves of CD3^+^ cells on OS (Overall Survival) with Collett’s Model for Selection of Covariates. (**F**) Cox Regression Curves of CD8^+^ cells on DFS (Disease Free Survival) with Collett’s Model for Selection of Covariates.

Higher PVI of CD3^+^ (*p* = 0.016) and CD8^+^ (*p* = 0.028) cells predicted better disease-free survival (DFS). Additionally, a positive tendency towards better DFS was noted for the PVI of CD20^+^ cells (*p* = 0.076). However, the PVI of CD66b^+^ cells had no influence on DFS (*p* = 0.521) (Figure 3 and Table S3). The PVI of CD3^+^ (*p* = 0.058), CD8^+^ (*p* = 0.297), CD20^+^ (*p* = 0.535), or CD66b^+^ (*p* = 0.616) cells did not influence overall survival (Figure S4 and Table S4).

Univariate analysis for each covariate was performed as shown in Table 2. Based on the Collett’s model selection approach, the variables CD3^+^ cells and Age (≥60 years) remained in Cox’s multivariate model, predicting overall survival (OS) as shown in Table S5 and Figure 3E. These results demonstrated that high and low amounts of CD3^+^ cells serve as an independent predictor of OS (*p* = 0.022). For DFS, the same selection process was used. Following the protocol, the variables gender and CD8^+^ cells remained after the selection, as shown in Table S6 and Figure 3D. The results clearly showed that CD8^+^ cells (*p* = 0.006), as well as gender (*p* = 0.003), were independent predictors for DFS.

### 2.4. Perivascular Infiltration Scoring (PVIS) Predicts Survival of All HCC Patients

**Figure 4:**
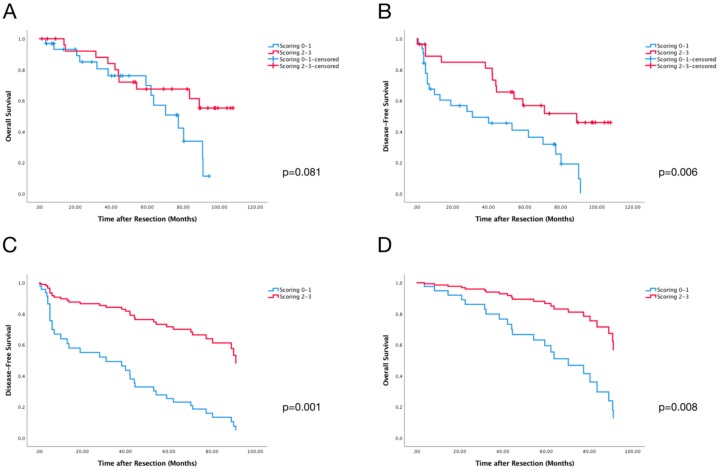
Kaplan-Meier Curves of Scoring on (**A**) overall survival (OS) and (**B**) disease-free survival (DFS). (**C**) Cox Regression Curves of Scoring on DFS with Collett’s Model for Selection of Covariates. (**D**) Cox Regression Curves of Scoring on OS with Collett’s Model for Selection of Covariates.

Based on the median values, each patient was given a binary score (1 as high and 0 as low) for each lymphocyte (CD3, CD8, and CD20). PVIS was calculated by summation of these binary scores. Patients were further divided into two groups (Low Group: PVIS 0-1, High Group: PVIS 2-3). Kaplan-Meier analysis of PVIS on overall survival showed a trend towards significant prognostic effect on OS (*p* = 0.081) (Figure 4A and Table S7). It should be stressed that PVIS (*p* = 0.006) had significant prognostic value for DFS (Figure 4B and Tables S8 and S9).

Finally, the Collett’s model selection approach was repeated, including PVIS, as mentioned above. The variables PVIS, cirrhosis, and age (≥60 years) eventually remained in Cox’s multivariate model, predicting overall survival as shown in Figure 4C and Table S9. In the multivariate analysis, PVIS showed significant prediction of OS (*p* = 0.008). For DFS, the variables PVIS and gender eventually remained after the selection process (Figure 4D and Table S10). Regarding PVIS (*p* = 0.001) and gender (*p* = 0.001), an independent predictive effect on DFS was found.

## 3. Discussion

In this study, we found for the first time that the PVI of CD3^+^ cells is an independent predictor of OS and the PVI of CD8^+^ cells is an independent predictor of DFS after the resection because of HCC. Furthermore, PVIS of CD3^+^, CD8^+^, and CD20^+^ cells is an independent predictor of both OS and DFS.

In general, previous studies have suggested that higher intratumoral infiltration of CD3^+^ cells predicts better OS or DFS after HCC resection [6,8,12,14,34]; the infiltration of CD8^+^ cells predicts better DFS or OS [6,8,9,10,11,12,13,15]. These studies did not, however, examine the specific PV location. 

Our study, using the recently established quantification of the tumor immune stroma (QTiS) algorithm, found that high perivascular infiltration of CD3^+^ cells is an independent predictor for favorable OS after curative resection of HCC. However, the multivariate procedure with the Collett’s model showed the independent influence of CD8^+^ cells on DFS but not OS. This lacking predictability of OS by CD8^+^ might be attributed to prompt postoperative treatments in cases of recurrence, as described in the results section. As soon as recurrences were detected during follow-up, the patients were admitted and evaluated for postoperative treatments including adjuvant therapies or even re-operations (as mentioned above).

B-cells have recently been recognized as an important player against tumor progression [35]. The tumor-infiltrating B-cell might not only directly induce cell death in tumor cells through antibody-independent mechanisms, but can also modulate T-cell responses. Conversely, B-cell activation, proliferation, and antibody production can also be inhibited by regulatory T-cells [36]. In several kinds of solid tumors, such as ovarian cancer, pancreatic cancer, lung cancer, and cervical cancer, the tumor-infiltrating B-cell has been widely associated with better outcome [21,37,38,39]. However, the prognostic significance of intratumoral infiltration of B-cells in HCC has only been shown in one study and failed in another [14,16]. In our results, although not significant, we witnessed a tendency towards better DFS with high perivascular infiltration of CD20^+^ cells.

In addition to adaptive immunity, innate immunity has been suggested to play a vital role in the progression of HCC. The CD66b^+^ neutrophils are the most common and abundant subpopulation of leukocytes and are considered as the initial battlefront of the defense against pathogens. In recent years, several studies have demonstrated that the peripheral neutrophil-to-lymphocyte ratio (NLR) negatively correlates with the survival of HCC patients [40,41]. However, very few studies have examined the role of the infiltration of neutrophils in HCC. Only one study investigated the infiltrating CD66^+^ cells and found that they correlated with worse DFS and OS [10]. Our results do not support the previously reported predictive outcome regarding the infiltration of CD66b^+^ cells [10]. Possible explanations for this could be the antibody heterogeneities or different analysis methods. Another possibility could be the etiological difference of the current cohort. There were only seven cases of hepatitis and 15 with cirrhosis, in comparison with over 90% cases that had hepatitis in the cohort measured in the study by Li and colleagues [10]. Neutrophils in tumor tissue can polarize to pro- or anti-tumorigenic subtypes depending on the tumor microenvironment, which varies greatly depending on the primary liver disease [42].

HCC is one of the most angiogenic solid tumors, similar to colorectal cancer [29,33]. Here, we introduced the perivascular region as a potential battlefront between tumor cells and immune cells. In the initial pattern review of our slides, we observed that high densities of intratumoral infiltration of immune cells were located near the vessels, with gradually decreasing densities in the tumor parenchyma. Thus, we speculated that extravasation from the vessels to the parenchyma is a major mode for infiltration of tumor-associated leukocytes. We have also confirmed that patients with curative resection could benefit from perivascular infiltration of immune cells. In a survival analysis, however, we could not show a difference of OS for perivascular infiltration alone. The influence on DFS was mixed regarding the detected infiltration patterns. While perivascular infiltration of CD8^+^ showed a significant advantage in DFS, perivascular CD20^+^ infiltration predicted significantly worse DFS. In this setting, it is suggested that the intratumoral neovascularization might have dual effects. It could allow potential metastatic cells, especially cancer stem cells, to escape from the primary tumor into circulation which could cause relapse or metastasis postoperatively [43]. Meanwhile, as a potential border to the host’s immune system, it also promotes immune cell infiltration which is indeed dependent on the network of blood vessels. Extravasation is mainly present in the post-capillary venules. The trafficking mechanism of immune cells in the tumor remains controversial. A previous study in breast cancer suggested an active ability of intratumoral high endothelial venules to recruit circulating lymphocytes. Thus, the densities of these vessels could even predict better survival [32]. In addition, Freeman MR et al. reported that the peripheral and tumor-infiltrating T-cell could synthesize vascular endothelial growth factor (VEGF) which is a mediator of neovascularization [44]. However, Huang H et al. showed in their study that VEGF suppresses T-cell infiltration through the inhibition of NF-κB-induced endothelial activation [45]. Additionally, the multikinase inhibitor, Sorafenib, was described to enhance the antitumor immunity in a murine model [46]. So, a deeper understanding of the mechanisms of intratumoral immune cell migration in conjunction with neovascularization is urgently needed and will be of great value for risk stratification.

Furthermore, we have developed a novel scoring to include the perivascular-infiltrating lymphocytes (PVIS). We are able to confirm that PVIS can independently predict both DFS and OS after HCC resection. It is easily practicable and might provide valuable information for HCC postresection surveillance.

## 4. Materials and Methods

### 4.1. Materials

Primary antibodies, anti-CD3 (ab5690), anti-CD8 (ab17147), anti-CD20 (ab78237), and anti-CD66b (ab197678) were purchased from Abcam Plc. (Cambridge, Cambs, UK). Secondary antibodies, Horse Anti-Rabbit IgG (BA-1100), and Horse Anti-Mouse IgG (BA-2000) were purchased from VECTOR Laboratories (Burlingame, CA, USA). Isotype controls against IgG were used as appropriate.

Avidin/Biotin Blocking Kit (SP-2001), ABC-AP (Alkaline phosphatase enzyme detection system, AK-5000), and ImmPACT Red Alkaline Phosphatase Substrate Kit (SK-5105) were purchased from VECTOR Laboratories (Burlingame, CA, USA).

### 4.2. Patients and Clinical Data

Tumor samples of 60 patients who underwent curative resection from November 2004 to October 2015 in our institution were asservated. All patients were clinically diagnosed according to EASL guidelines and confirmed histologically after the resection [47]. Tissue samples and annotated data were obtained, and experimental procedures were performed within the framework of the non-profit foundation HTCR, including the informed patient’s consent [48]. All tumor samples were anonymously coded. This study was approved by the institutional review board of the Ludwig-Maximilians University in Munich (EK266-16, EK258-16).

The following were examined during clinical routine and collected in our database: epidemiological data and clinical characteristics including gender, age, hepatitis, the presence of cirrhosis, tumor number, microvascular and macrovascular invasion, Milan staging; routine lab values relevant for liver surgery including serum alpha-fetoprotein, bilirubin, albumin, alanine transaminase (ALT), aspartate transaminase (AST), activated partial thromboplastin time (APTT), creatinine, c-reactive protein (CRP), leukocytes count, and the platelet count of all patients [47].

### 4.3. Immunohistochemical Staining

Four-micrometer sections of paraffin blocks were cut for IHC staining. Briefly, IHC staining was performed as described previously [49]. Dewaxing and hydration were done with xylene, ethanol, and distilled water. Antigens were retrieved using citrate buffer (pH 6.0) or EDTA solution (pH 8.0). Following the incubation with antibodies against CD3, CD8, CD20, and CD66b and incubation with secondary antibodies, ABC-AP was used for staining. Counterpart staining was performed with Hemalaun. Negative and isotype controls were used as appropriate.

### 4.4. Analysis of Immunohistochemical Staining

Hotspots were defined as areas with the highest infiltration density. An algorithm for quantification of the tumor immune stroma (QTiS) was used [50]. Hotspots were chosen under low power field (40×). However, in this analysis, hotspot search was limited to the perivascular regions inside the tumor. Vessels in the intratumoral stroma were assessed according to their typical morphology. In the case of an even infiltration pattern, perivascular hotspots were defined by the fact that at least three of the four analyzed cell groups exhibited infiltration around that area. In accordance with the QTiS algorithm, we analyzed the mean amount of three perivascular hotspot regions. This was only possible if enough infiltration, and thereby hotspots, were present. The pictures were captured under high-power magnification (Olympus BX40F with Carl Zeiss AxioCam MRc5, 200×) with the selected vessel in the center of the shot. The numbers of positive cells per image were counted using ImageJ software (Version 1.51h, National Institutes of Health; Bethesda, MD, USA) with threshold adjusting and were recorded in our database for further analysis.

### 4.5. Statistical Analysis

Continuous numbers were presented as mean ± SD or as median (Interquartile Range) appropriately. First, normality was examined by the Shapiro-Wilk test. Then they were compared using the independent *t*-test if normally distributed or using the Mann-Whitney u-test if they were not normally distributed. The χ^2^-test or Fisher’s exact test was applied to compare contingency variables. Pearson’s r values were calculated to evaluate the correlation. The median values of immune cell amounts were calculated to classify the patients into high (>medians) or low (≤medians) groups for survival analysis. The log-rank test was used in univariate survival analysis. The Cox model was used in multivariate survival analysis. The Collett’s model selection approach was applied for covariate screening. In this stepwise evaluation, covariates with p1 < 0.200 in the univariate analysis were included in the multivariate analysis. After inclusion, these covariates were eliminated if p2 was greater than 0.100. To double-check all other covariates, a forward selection multivariate analysis with non-predictive variables was performed (p3). If the variables achieved p3 < 0.100, they could be included in the final multivariate analysis. For the final step, covariates were independently predictive if p4 < 0.050 [51]. DFS was defined as the period after resection without recurrence. OS was defined as the time from resection to death. All statistics were performed using Prism (Version 7.00, GraphPad, La Jolla, CA, USA) and SPSS statistics software (Version 24.0, IBM, Armonk, NY, USA). In general, *p* < 0.050 was considered statistically significant.

## 5. Conclusions

In conclusion, our study found that PV-infiltrating CD3^+^ cells are an independent predictor of better OS, and that CD8^+^ cells independently predict prolonged DFS. Our novel scoring of perivascular-infiltrating lymphocytes can independently predict both DFS and OS. With this simple prediction tool, follow-up resources can be guided towards patients with a higher propensity for recurrences.

## Figures and Tables

**Table 1:** Demographics of the Study Population.

Variables	Results
Gender (Male/Female)	49 (81.7%)/11 (18.3%)
Age (Years) (Median (IQR))	66.00 (16.00)
Hepatitis (HBV/HCV)	7 (11.7%)/0 (0.0%)
Cirrhosis	15 (25.0%)
AFP (ng/mL) (Median (IQR))	12.60 (153.00)
Tumor multiplicity	9 (15.0%)
Microvascular invasion	18 (30.0%)
Macrovascular invasion	6 (10.0%)
Beyond Milan Criteria	47 (78.3%)
Bilirubin (mg/dL) (Median (IQR))	0.70 (0.30)
Albumin (mg/dL) (Median (IQR))	43.00 (5.00)
ALT (U/L) (Median (IQR))	42.00 (33.00)
AST (U/L) (Median (IQR))	45.00 (37.00)
APTT (s) (Median (IQR))	28.50 (5.25)
Creatinine (mg/dL) (Median (IQR))	1.00 (0.20)
CRP (mg/L) (Median (IQR))	6.00 (16.00)
Leukocytes (10^3^/µL) (Median (IQR))	7246.67 (2094.03)
Platelets (10^3^/µL) (Median (IQR))	222.00 (120.50)

Abbreviations: IQR, Interquartile Range; HBV, hepatitis B; HCV, hepatitis C; AFP: serum alpha-fetoprotein; BCLC: Barcelona Clinic Liver Cancer; AJCC: American Joint Committee on Cancer; ALT: alanine transaminase; AST: aspartate transaminase; APTT: activated partial thromboplastin time; CRP: C-reactive protein.

**Table 2:** Univariate Analysis of All Factors on OS and DFS in all Hepatocellular Carcinoma (HCC) Patients (*n* = 60).

Variables	Overall Survival				Disease-free Survival			
	HR	95% CI		*p*	HR	95% CI		*p*
Gender	1.243	0.781	1.978	0.355	2.312	1.112	4.808	**0.021**
Age (≥60 years)	0.440	0.150	1.291	0.124	1.496	0.754	2.971	0.246
Hepatitis	0.237	0.077	0.732	**0.007**	2.451	0.932	6.447	**0.059**
Cirrhosis	0.525	0.219	1.256	0.141	0.843	0.399	1.779	0.652
AFP (≥20 ng/mL)	0.913	0.389	2.145	0.835	0.638	0.322	1.266	**0.195**
AFP (≥400 ng/mL)	1.798	0.420	7.706	0.423	1.364	0.478	3.889	0.560
Tumor Multiplicity	1.521	0.355	6.517	0.570	0.818	0.339	1.973	0.653
Microvascular Invasion	0.677	0.272	1.690	0.401	0.708	0.346	1.447	0.338
Macrovascular Invasion	0.702	0.201	2.457	0.578	0.618	0.232	1.644	0.328
Beyond Milan	0.553	0.073	4.187	0.560	0.272	0.037	2.006	**0.169**
Low CD3^+^ Cells	2.196	0.956	5.046	**0.057**	2.277	1.145	4.527	**0.016**
Low CD8^+^ Cells	1.531	0.684	3.425	0.297	2.074	1.064	4.043	**0.028**
Low CD20^+^ Cells	1.291	0.574	2.904	0.535	0.549	0.279	1.079	**0.076**
Low CD66b^+^ Cells	1.228	0.550	2.744	0.616	1.239	0.641	2.394	0.521

Abbreviations: HR: Hazard ratio; CI: confidence interval; *p*: *p*-value; AFP: serum alpha-fetoprotein; HCC: Hepatocellular Carcinoma. The *p*-values under 0.200 are bolded.

## Supplementary Materials

The following are available online at http://www.mdpi.com/2072-6694/10/10/389/s1, Figure S1. No Infiltration of CD3+ (A), CD8+ (B), CD20+ (C), and CD66b+ (D) Cells under 100x Magnifications; Figure S2. Evenly Distributed Infiltration Patterns of CD3+ (A), CD8+ (B), CD20+ (C), and CD66b+ (D) Cells under 100x Magnifications; Figure S3. Correlations among perivascular-infiltrating CD3+, CD8+, CD20+, and CD66b+ cells; Figure S4. Kaplan-Meier Curves of CD3+ (A), CD8+ (B), CD20+ (C), and CD66b+ (D) Cells on Overall Survival; Table S1. Number of Cases in Three Different Patterns; Table S2. Relationships and Correlations between Perivascular Infiltration with Clinical Parameters; Table S3. Estimated Cumulative Proportion of Disease-free Survival Regarding CD3+, CD8+, CD20+, and CD66b+ cells; Table S4. Estimated Cumulative Proportion of Overall Survival Regarding CD3+, CD8+, CD20+, and CD66b+ cells; Table S5. Cox’s Multivariate Model for Predicting Overall Survival with Collett’s Model for Selection of Covariates; Table S6. Cox’s Multivariate Model for Predicting Disease-Free Survival with Collett’s Model for Selection of Covariates; Table S7. Estimated Cumulative Proportion of Overall Survival Regarding Scoring; Table S8. Cox’s Multivariate Model for Predicting Overall Survival with Collett’s Model for Selection of Covariates; Table S9. Cox’s Multivariate Model for Predicting Disease-Free Survival with Collett’s Model for Selection of Covariates; Table S10. Estimated Cumulative Proportion of Disease-free Survival Regarding Scoring.
