# Supplementary material for: Perivascular Tumor-Infiltrating Leukocyte Scoring for Prognosis of Resected Hepatocellular Carcinoma Patients
Source: Cancers (Basel). 2018 Oct 18;10(10):389. doi: 10.3390/cancers10100389 (PMC6210365; doi:10.3390/cancers10100389)
Supplement: Supplementary file 1 [file cancers-10-00389-s001.pdf]

Supplementary Materials: Perivascular Tumor-Infiltrating Leukocyte Scoring for Prognosis of Resected Hepatocellular Carcinoma Patients

Markus Bo Schoenberg, Jingcheng Hao, Julian Nikolaus Bucher, Rainer Christoph Miksch, Hubertus Johann Wolfgang Anger, Barbara Mayer, Julia Mayerle, Jens Neumann, Markus Otto Guba, Jens Werner and Alexandr V Bazhin

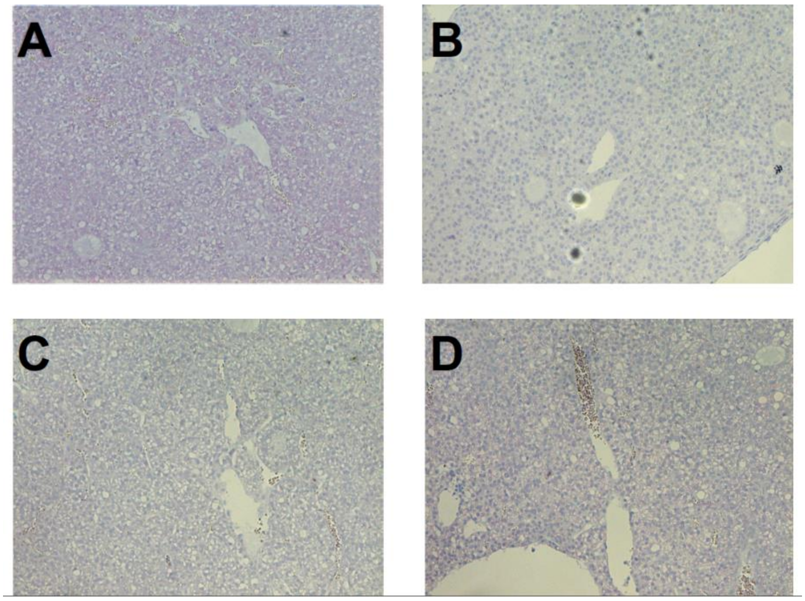

Figure S1. No Infiltration of $CD3^+$ (**A**), $CD8^+$ (**B**), $CD20^+$ (**C**), and $CD66b^+$ (**D**) Cells under 100× Magnifications.

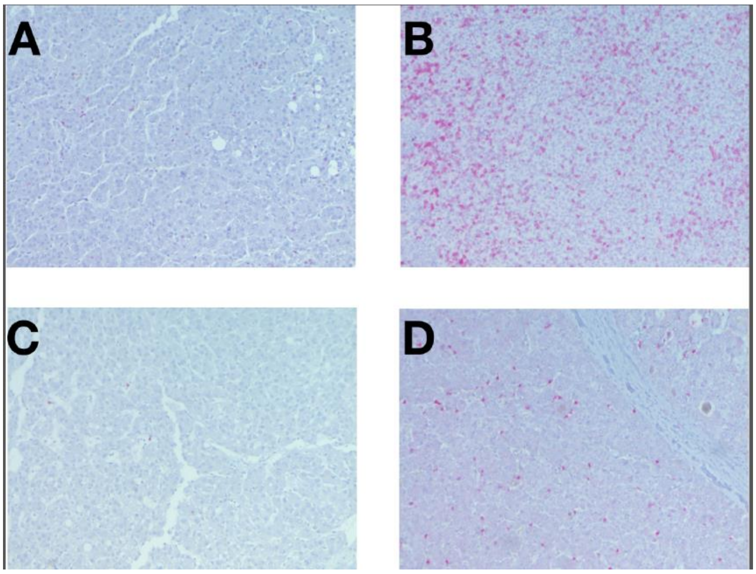

Figure S2. Evenly Distributed Infiltration Patterns of $CD3^+$ (**A**), $CD8^+$ (**B**), $CD20^+$ (**C**), and $CD66b^+$ (**D**) Cells under 100× Magnifications.

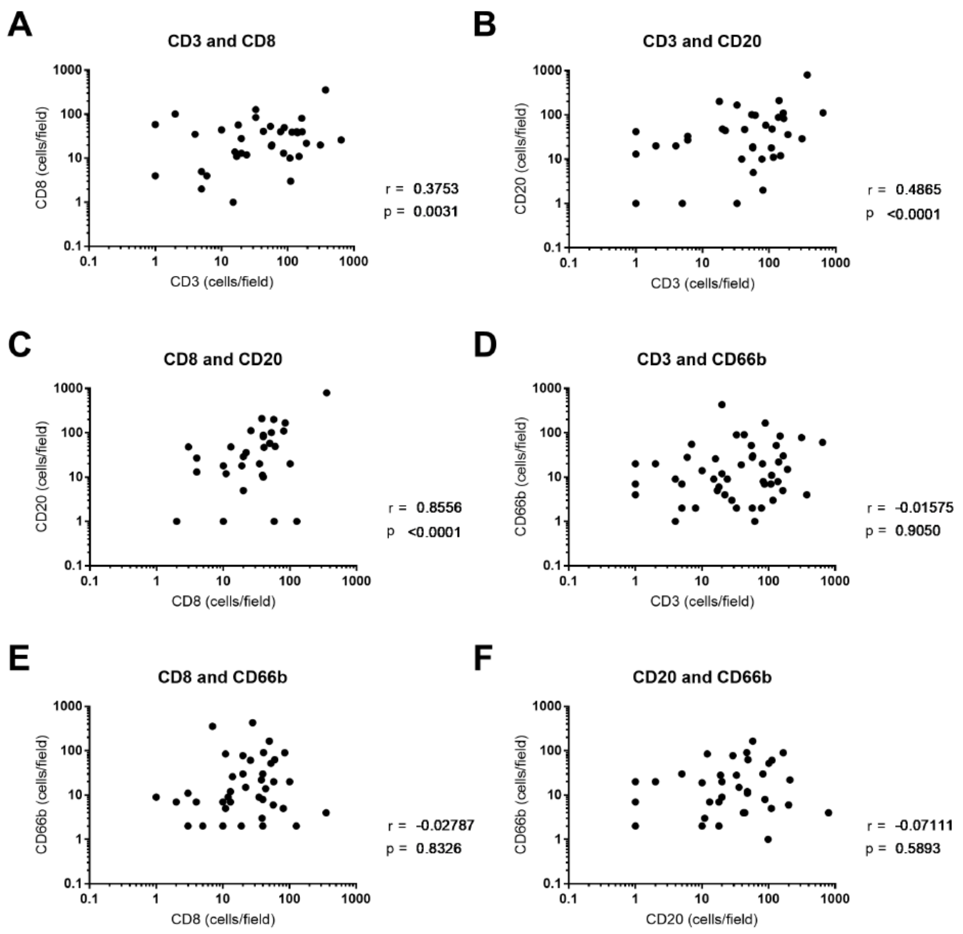

Figure S3. Correlations among perivascular-infiltrating CD3+, CD8+, CD20+, and CD66b+ cells. (**A**) Correlation between CD3+ andCD8+ cells; (**B**) Correlation between CD3+ andCD20+ cells; (**C**) Correlation between CD8+ andCD20+ cells; (**D**) Correlation between CD3+ andCD66b+ cells; (**E**) Correlation between CD8+ andCD66b+ cells; (**F**) Correlation between CD20+ andCD66b+ cells.

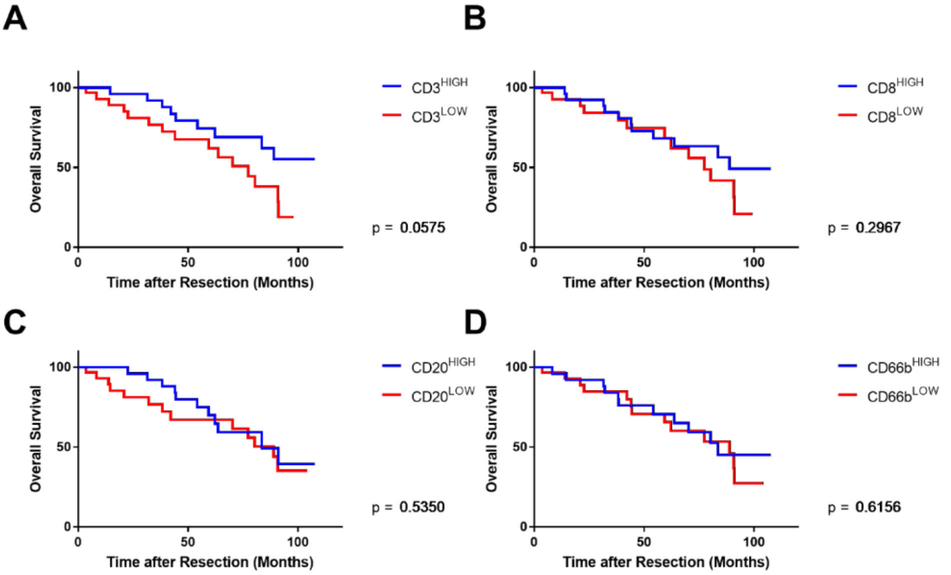

Figure S4. Kaplan–Meier Curves of CD3+ (A), CD8+ (B), CD20+ (C), and CD66b+ (D) Cells on Overall Survival.

Table S1. Number of Cases in Three Different Patterns.

	Negative	Evenly Distribution	Perivascular Distribution

CD3	9(15.0%)	16(26.7%)	35(58.3%)
CD8	20(33.3%)	10(16.7%)	30(50.0%)
CD20	23(38.3%)	10(16.7%)	27(45.0%)
CD66b	2(3.3%)	24(40.0%)	34(56.7%)

Table S2. Differences in demographic and clinical laboratory values in dependence of high vs. low perivascular infilatration of $CD3^+$, $CD8^+$, $CD20^+$, $CD66b^+$ leukocytes.

**Variables**		**CD3**			**CD8**			**CD20**			**CD66b**		
		**Low**	**High**	***p***	**Low**	**High**	***p***	**Low**	**High**	***p***	**Low**	**High**	***p***
Gender	Male	25	24	1.000	24	25	1.000	25	23	0.745	25	24	1.000
	Female	5	6		6	5		5	6		6	5	
Age (Years)		65.50 (17.00)	67.00 (15.00)	0.796	64.00 (20.00)	67.50 (14.00)	0.438	65.00 (18.00)	66.00 (13.00)	0.986	68.00 (16.00)	63.00 (23.00)	0.593
AFP (ng/ml)		8.75 (80.48)	18.20 (350.75)	0.495	7.60 (130.65)	14.10 (232.25)	0.887	13.40 (199.85)	7.10 (106.40)	0.895	14.45 (104.83)	9.80 (1391.50)	0.684
HBV	**Yes**	5	2	0.426	5	2	0.420	5	2	0.426	4	3	1.000
	**No**	23	23		22	24		23	23		25	21	
ETOH	**Yes**	6	5	1.000	7	4	0.505	6	5	1.000	6	5	1.000
	**No**	22	21		21	22		22	21		23	20	
Cirrhosis	**Yes**	8	7	1.000	7	8	0.764	8	7	1.000	8	7	1.000
	**No**	20	19		21	18		20	19		21	18	
Tumor Number	**Single**	24	26	0.472	26	24	0.472	26	24	1.000	25	25	1.000
	**Multiple**	6	3		3	6		5	4		5	4	
Microvascular	**Yes**	12	6	0.155	8	10	0.773	8	10	0.566	10	8	0.777
Invasion	**No**	16	20		19	17		20	16		18	18	
Macrovascular	**Yes**	3	3	1.000	4	2	0.388	2	4	0.417	4	2	0.417
Invasion	**No**	20	23		18	25		23	20		20	23	
UICC Stage	**I-II**	8	5	0.339	8	5	0.339	6	7	1.000	4	9	0.196
	**III-IV**	16	21		16	21		18	19		21	16	
Milan Criteria	**In**	2	3	0.662	2	3	1.000	3	2	1.000	2	3	1.000
	**Out**	25	22		24	23		25	22		24	23	
BCLC Stage	**A**	5	2	0.232	3	4	1.000	3	4	1.000	3	4	1.000
	**B**	16	21		19	18		19	18		18	19	
BRC Criteria	**In**	8	14	0.076	9	13	0.376	10	12	0.768	13	9	0.554
	**Out**	15	8		13	10		12	11		11	12	

BILI	0.70 (0.40)	0.70 (0.30)	0.836	0.60 (0.30)	0.70 (0.33)	0.976	0.70 (0.33)	0.70 (0.30)	0.635	0.60 (0.23)	0.70 (0.50)	0.391
ALB	44.00 (5.00)	43.00 (7.25)	0.425	41.00 (5.00)	44.00 (5.00)	**0.004**	42.00 (5.50)	44.00 (5.00)	**0.019**	43.00 (6.00)	44.00 (3.00)	0.706
ALT	45.00 (40.00)	40.00 (31.00)	0.780	46.00 (53.25)	34.00 (31.50)	0.060	43.00 (32.75)	42.00 (35.00)	0.798	44.00 (21.75)	30.00 (41.00)	0.254
AST	52.50 (42.00)	41.00 (20.00)	0.419	52.00 (39.50)	38.00 (30.00)	0.058	46.00 (33.25)	41.00 (34.00)	0.329	46.00 (25.75)	41.00 (47.00)	0.717
APTT	28.00 (6.00)	29.00 (6.00)	0.755	28.00 (7.50)	29.00 (4.50)	0.537	28.00 (7.50)	29.00 (5.50)	0.690	27.00 (7.00)	29.00 (5.00)	0.815
CREA	1.00 (0.23)	1.00 (0.25)	0.793	0.95 (0.30)	1.00 (0.22)	0.373	0.90 (0.30)	1.00 (0.29)	0.155	1.00 (0.28)	1.00 (0.25)	0.793
CRP	7.00 (19.25)	5.00 (12.50)	0.653	7.00 (31.00)	5.00 (9.00)	0.849	5.00 (27.00)	6.50 (10.75)	0.455	6.00 (14.00)	5.50 (20.50)	0.415
WBC （mean±SD）	7230±2098	7263±2126	0.951	7179 ±2229	7315 ±1986	0.804	7210 ±2032	7286 ±2194	0.889	7143 ±1695	7357 ±2477	0.700
PLATELETS	225.00 (192.75)	211.50 (114.50)	0.387	214.50 (206.75)	222.00 (105.75)	0.348	226.00 (192.00)	214.00 (121.00)	0.311	200.00 (110.00)	265.00 (157.00)	0.294

Abbreviations: AFP: serum alpha-fetoprotein; ALT: alanine transaminase; AST: aspartate transaminase; APTT: activated partial thromboplastin time; CRP: C-reactive protein. Normally distributed data was compared with the T-test and not normally distributed data with Mann-Whitney u test. Contingency data was compared using the Fisher's exact test.

Table S3. Estimate Cumulative Proportion of Disease-free Surviving Regarding $CD3^+$, $CD8^+$, $CD20^+$, and $CD66b^+$ cells.

Groups	1-year	3-year	5-year	8-year
$CD3^{LOW}$	69.33%	51.57%	39.50%	6.83%
$CD3^{HIGH}$	82.03%	82.03%	57.55%	46.16%
$CD8^{LOW}$	64.39%	56.35%	42.91%	7.42%
$CD8^{HIGH}$	86.31%	75.95%	53.41%	42.48%
$CD20^{LOW}$	66.93%	52.60%	40.71%	17.81%
$CD20^{HIGH}$	84.86%	81.00%	50.72%	36.07%
$CD66b^{LOW}$	75.68%	64.87%	45.79%	15.18%
$CD66b^{HIGH}$	75.14%	71.38%	49.90%	32.34%

Table S4. Estimate Cumulative Proportion of Overall Surviving Regarding $CD3^+$, $CD8^+$, $CD20^+$, and $CD66b^+$ cells.

Groups	1-year	3-year	5-year	8-year
$CD3^{LOW}$	92.95%	76.72%	61.99%	19.02%
$CD3^{HIGH}$	100%	91.83%	74.35%	55.23%
$CD8^{LOW}$	92.64%	84.22%	68.35%	20.97%
$CD8^{HIGH}$	100%	84.62%	68.15%	49.22%
$CD20^{LOW}$	93.05%	76.72%	67.05%	35.20%
$CD20^{HIGH}$	100%	92.00%	70.00%	39.49%
$CD66b^{LOW}$	96.67%	84.73%	65.57%	27.48%
$CD66b^{HIGH}$	96.00%	84.00%	70.57%	45.12%

Table S5. Cox Multivariate Model for Predicting Overall Survival with Collett´s Model for Selection of Covariates.

Variables	HR	95% CI		*p*
Age (≥60 years)	0.332	0.109	1.012	0.053
$CD3^+$ Cells	2.754	1.158	6.548	0.022

Abbreviations: HR: Hazard ratio; CI: confidence interval.

Table S6. Cox Multivariate Model for Predicting Disease-Free Survival with Collett´s Model for Selection of Covariates.

Variables	HR	95% CI		*p*
Gender	3.405	1.517	7.645	0.003
$CD8^+$ Cells	2.819	1.353	5.876	0.006

Abbreviations: HR: Hazard ratio; CI: confidence interval.

Table S7. Estimate Cumulative Proportion of Overall Surviving Regarding Scoring.

Groups	1-year	3-year	5-year	8-year
Scoring: 0-1	93.15%	80.57%	69.76%	11.27%
Scoring: 2-3	100%	88.00%	67.50%	55.23%

Table S8. Estimate Cumulative Proportion of Disease-free Surviving Regarding Scorings of Scoring.

Groups	1-year	3-year	5-year	8-year
Scoring: 0-1	63.95%	49.26%	40.93%	0%
Scoring: 2-3	88.71%	84.86%	56.83%	45.92%

Table S9. Cox Multivariate Model for Predicting Overall Survival with Collett´s Model for Selection of Covariates.

Variables	HR	95% CI		*p*
Age	0.359	0.117	1.102	0.073
Cirrhosis	0.430	0.175	1.055	0.065
Scoring	3.624	1.406	9.338	0.008

Abbreviations: HR: Hazard ratio; CI: confidence interval.

Table S10. Cox Multivariate Model for Predicting Disease-Free Survival with Collett´s Model for Selection of Covariates.

Variables	HR	95% CI		*p*
Gender	4.364	1.861	10.234	0.001
Scoring	4.113	1.841	9.190	0.001

Abbreviations: HR: Hazard ratio; CI: confidence interval.
